# Milk as a Factor in the Causation of Disease

**Published:** 1896-04

**Authors:** John M. Parker

**Affiliations:** Haverhill, Mass.


### THE JOURNAL OF COMPARATIVE MEDICINE AND VETERINARY ARCHIVES.

VOL. XVII. *APRIL*, 1896. No. 4.

### MILK AS A FACTOR IN THE CAUSATION OF DISEASE.

BY JOHN M. PARKER, D.V.S.,
HAVERHILL, MASS.

IN considering this subject I shall not attempt to do more than skim the surface. The subject is so large that I have been unable to do more than touch on some of the more important parts. There are several points of great interest closely connected with this subject, such as the influence of food-stuffs on the healthfulness of milk, that I have not touched upon, and which I hope to be able to take up at some future time.

Milk is the natural food for infants. In a perfect state it is the most perfect food we know, and, of course, it follows that it is of the utmost importance that it be procured and placed on the market in as perfect a condition as possible. And here the study of bacteriology has been of the utmost importance to the dairy-farmer. Housewives used to dread the thunderstorm because the condition of the atmosphere was supposed to exert a peculiar influence on milk, causing it to sour. The study of bacteriology has shown the fallacy of this theory. The warm weather preceding the storm favors the development of germ-life, which finds its way into the milk, causing it to sour and curdle. To prevent this from taking place, and to keep milk pure and sweet, it is only necessary to prevent the entrance or development of the various forms of bacteria; if this is done milk will keep almost indefinitely. Practically, however, it is almost impossible to keep milk perfectly free from germ-life, but with a little care the number can be greatly reduced.

As a rule the greatest number of bacteria find their way into milk during milking. The milker usually rests his head on the cow's flank immediately above the pail, and of necessity dirt and scurf and hairs from the cow find their way into the milk. The cow has possibly been standing in a mud-hole in the pasture, and the udder, body, flank, and tail are probably covered with dirt and filth and decaying animal and vegetable matter, and, of course, the spores and germs from the grasses, marshes, and stagnant water are brushed and shaken off during milking and drop into the milk. One has only to take a casual glance at the strainer after the milk has passed through to see straws and hairs and scales from sore teats or udders, along with feces and dirt and filth of all kinds, all of which are teeming with germ-life, and he will realize what necessity there is for a change of method to keep pace with our increased knowledge on the subject.

Contamination, however, does not only come from the cow, but the milker's hands and clothing, as well as pails, strainers, receptacles for holding and cooling milk, milk-jars, and skimmers. All should be sterilized. It is not sufficient to rinse out a pail with warm water. Pails should be washed with soda solution and then with boiling water, and if there are any seams in the pail these should be scrubbed with a brush (all seams in the pail, without exception, should be soldered, otherwise the seam itself will be a receptacle for micro-organisms). Milkers' hands should be thoroughly washed with soap and warm water, and, instead of any dirty clothes being used for milking, clean overalls should invariably be used. The udder should also be thoroughly washed before milking. In this way the micro-organisms that are not washed off will adhere to the damp surface, and will not be so likely to fall off into the milk. Cows should also be groomed and curried each day as thoroughly as a horse. These are no idle fancies, but are well-established facts, well illustrated by several investigators, notably by Dr. P. H. Bryce, of Ontario. In Dr. Bryce's experiments, gelatin-plate cultures were poured, ½ c.c. of milk being added for each culture. In *three days*, at a temperature of 66.70° F., the various plates gave the following results:

Plate I. Direct from the cow; no precaution except that the milk was received into sterilized test-tubes; 15 micro-organisms per c.c.

Plate II. Pail in stable receiving milk from different cows; milk strained through a cloth; 720 micro-organisms per c.c.

Plate III. Milk from cooling apparatus after cooling; 884 micro-organisms per c.c.

Plate IV. Milk from bottles immediately after filling; 1640 micro-organisms per c.c.

These experiments plainly show the greatest source of contamination. In the first plate, milking directly into a test-tube, after three days at a temperature of 66.70° F., there were only 15 micro-organisms per c.c. This milk came directly from the cow into a sterilized test-tube, and, of course, there was no outside contamination. In the next plate there were 720 germs in each c.c. after being milked into a pail and strained through a cloth. After cooling there were 884, and after filling into bottles there were 1640. As the milk came into contact with more surface the numbers increased rapidly, and this, remember, on a farm where the milk was handled with unusual care as regards cleanliness.

Experiments conducted by Dr. Peters show another occasional source of contamination, especially in cows after standing in stagnant water or lying on dirty floors. These experiments were carried out under careful antiseptic precautions. Clean suits were put on; the cows' udders, teats, flanks, sides, groins, and abdomen were washed with bichloride solution; they were milked into sterile bottles, four cows being used in the experiments.

Table marked I represents milk in first half of milking and drawn by hand directly into sterile bottles.

No. II. represents milk drawn through sterile canula into bottle.

Nos. III. and IV. represent milk drawn by hand after more than half the udder had been emptied.

The columns marked A, B, C, D, represent the milk from the four cows, the figures showing the number of colonies of bacteria in each plate:

| | A. | B. | C. | D. |
|---|---|---|---|---|
| I. | 141 | 167 | 19 | 53 |
| II. | 0 | 0 | 1 | 2 |
| III. | 0 | 6 | 0 | 0 |
| IV. | 0 | 0 | 1 | 2 |

It will be noticed that in the first milk drawn from each cow there were a number of colonies of bacteria, while the later portion of the milking was practically free from bacteria, the milk being drawn into sterile bottles.

It would seem, then, that as a matter of practical importance it would be well to strip the teat once or twice before milking into a sterilized pail. In this way the micro-organisms which evidently find their way into the milk-duct would be washed out. It also emphasizes the importance of cleanly surroundings for cattle, for we find that the cows referred to in Bryce's experiments were handled with unusual care as regards cleanliness, with the evident result that the first of the milk was not contaminated, as in Dr. Peters's experiments.

Not only should the greatest care be exercised as to cleanliness, however, but, after aërating the milk to rid it of bad odors, it should be rapidly cooled to about 41° F., the cooling of milk preventing or retarding the growth of the various micro-organisms contained in the milk. To again quote from Dr. Bryce: "Milk that had been kept in a cool place, and then showed 10,000 micro-organisms per c.c., was afterward allowed to stand in a warm room some six hours, and during this time the bacteria increased from 10,000 to 1,000,000 per c.c."

Experiments by Feer showed the same results: "In examining, a few hours after milking, milk that had been supplied to a children's hospital, he found in winter 50 to 70,000 bacteria per c.c. In summer, under the same conditions, they averaged 300,000, and after standing a few hours at ordinary temperature they multiplied to 14,000,000." (*Annual of Medical Sciences.*)

These experiments show the importance of cleanliness and of keeping the milk at a low temperature, not only at the dairy-farm, but after delivery to the customer. Many a farmer gets blamed for delivering impure or sour milk, when no one is at fault but the ignorant or careless housekeepers. Want of knowledge or want of appreciation of the importance of absolute cleanliness in the care of milk is perhaps one of the greatest, though unrealized, difficulties that health authorities will have to contend against.

Of all infantile disorders cholera infantum, or milk-diarrhœa, is by far the most fatal, and thousands of children die every summer from this cause alone. In referring to milk-diarrhœa, Keating says: "Its character and type are modified by three important factors: 1, climate or season; 2, social condition; 3, food. It is not now the belief that heat *per se* causes diarrhœa. It is only a powerful, indirect factor, lowering the vitality of the patient and favoring fermentative changes in the milk.

It is to the feeding-bottle and its contents that attention must be specially directed in the prevention of autumnal diarrhœa."

It only occurs in hot weather. A temperature of 60° F. or over favors the development and multiplication of the various micro-organisms that are the prime cause of this disease. There is no specific micro-organism present, but the presence of all toxicogenic bacteria (or bacteria that give off poisonous gases) in milk or food of any kind must be avoided. Infants fed exclusively on sterile foods are not liable to be attacked, and various methods have been tried to prevent the development of these organisms in milk. The principal of these are the Pasteurization and the sterilization of milk, and in European countries and in the West these methods are adopted quite extensively.

The Pasteurization of milk consists in the placing of milk in sterile bottles or jars and exposing them to a temperature of from 150° F. to 180° F. Milk treated in this way, cooled to 41° F., and kept at a temperature below 60° F. will keep sweet and pure for several days, and "sweet milk may be heated to 180° F. without any coagulation or burning or any material change taking place." (*Year-Book, Department of Agriculture,* 1895.) To Pasteurize milk with the best results, however, milk must be fresh. It should be perfectly pure, and as free as possible from bacteria. Where there are any great number of micro-organisms present, and where there is the least tendency to sour, even when so slight as not to be perceptible to taste, the heat will coagulate the albumin, rendering it more or less indigestible.

All germs are not destroyed at 180° F., however, and some even multiply at that temperature. To totally destroy all germs sterilizing at a temperature of 230° F. is required. On the other hand, that amount of heat would cause a coagulation of casein, and would injure the taste and appearance of the milk, but all germs and spores can be destroyed by interrupted heating, *i. e.*, by submitting the milk to a temperature of 180° F. and cooling, repeating the process for five or six consecutive days. The rapid change of temperature from hot to cold favors the keeping qualities of the milk by either killing the bacteria or lessening their vitality so that a long time is required before they can begin to multiply. (*Year-Book, Department of Agriculture*, 1896.)

It must be remembered, however, that the Pasteurization or sterilization of milk is not of itself an advantage to milk. It is undertaken simply for the purpose of destroying the activity of that which is foreign to milk and which finds its way into milk either during or after milking. Milk from healthy cows is of itself absolutely pure, and but for the entrance of foreign organisms would keep indefinitely. It follows, then, that an effort should be made to prevent the entrance of these foreign matters. Much can be done in this way, and for their own protection dairymen ought to do as much as possible and go as far as practicable in this direction.

(To be concluded in the May number.)

